# Supplementary material for: Characterization of the Complete Mitochondrial Genome of the Elongate Loach and Its Phylogenetic Implications in Cobitidae
Source: Animals (Basel). 2023 Dec 13;13(24):3841. doi: 10.3390/ani13243841 (PMC10740543; doi:10.3390/ani13243841)
Supplement: Supplementary file 1 [file animals-13-03841-s001.zip › Supplementary Tables.pdf]

Table S1. Non-synonymous substitution rate of mitochondrial genes in Leptobotia species

Species	*Leptobotia mantschurica*	*Leptobotia taeniops*	*Leptobotia microphthalma*	*Leptobotia rubrilabris*	*Leptobotia punctata*	*Leptobotia pellegrini*	Average
nd4	0.0166	0.0124	0.0110	0.0146	0.0101	0.0145	0.0132
nd5	0.0203	0.0202	0.0153	0.0143	0.0150	0.0177	0.0171
nd6	0.0087	0.0060	0.0091	0.0030	0.0061	0.0091	0.0070
cytb	0.0065	0.0054	0.0041	0.0081	0.0068	0.0041	0.0058
nd1	0.0081	0.0082	0.0066	0.0114	0.0115	0.0098	0.0093
nd2	0.0265	0.0179	0.0119	0.0134	0.0178	0.0120	0.0166
cox1	0.0078	0.0029	0.0049	0.0049	0.0083	0.0078	0.0061
cox2	0.0066	0.0022	0.0036	0.0066	0.0022	0.0022	0.0039
atp8	0.0075	NA	0.0151	NA	NA	0.0075	0.0100
atp6	0.0158	0.0113	0.0135	0.0135	0.0137	0.0091	0.0129
cox3	0.0095	NA	NA	NA	0.0019	NA	0.0057
nd3	0.0089	0.0086	0.0089	NA	0.0045	0.0089	0.0080
nd4l	0.0055	0.0055	NA	NA	NA	NA	0.0055
**Average**	0.0114	0.0092	0.0094	0.0100	0.0089	0.0093	

Table S2. Synonymous substitution rate of mitochondrial genes in Leptobotia species

Species	*Leptobotia mantschurica*	*Leptobotia taeniops*	*Leptobotia microphthalma*	*Leptobotia rubrilabris*	*Leptobotia punctata*	*Leptobotia pellegrini*	Average
nd4	0.3072	0.2587	0.1378	0.2807	0.2312	0.2810	0.2494
nd5	0.3390	0.2061	0.1508	0.2192	0.1948	0.2620	0.2286
nd6	0.3892	0.2009	0.1454	0.1851	0.2136	0.2839	0.2363
cytb	0.3130	0.2249	0.1558	0.1944	0.2038	0.2119	0.2173
nd1	0.2916	0.2499	0.1942	0.2269	0.2008	0.2503	0.2356
nd2	0.2375	0.2222	0.1452	0.2249	0.2176	0.2187	0.2110
cox1	0.3102	0.2498	0.1397	0.1906	0.1935	0.2488	0.2221
cox2	0.1696	0.1562	0.1393	0.1530	0.1376	0.1815	0.1562
atp8	0.2238	0.1930	0.1062	0.3132	0.2284	0.2686	0.2222
atp6	0.3279	0.2489	0.1982	0.2665	0.2852	0.2965	0.2705
cox3	0.2624	0.1976	0.1230	0.2050	0.1981	0.1609	0.1912
nd3	0.3510	0.2059	0.1736	0.1592	0.2136	0.2410	0.2241
nd4l	0.2358	0.1912	0.1103	0.1924	0.1234	0.2873	0.1901
**Avergae**	0.2891	0.2158	0.1476	0.2162	0.2032	0.2456	

Table S3. The ratio of the number of nonsynonymous substitutions per nonsynonymous site (Ka) to the number of synonymous substitutions per synonymous site (Ks) of mitochondrial genes in *Leptobotia* species.

Species	*Leptobotia*	*Leptobotia*	*Leptobotia*	*Leptobotia*	*Leptobotia*	*Leptobotia*	Average

	mantschurica	*taeniops*	*microphthalma*	*rubrilabris*	*punctata*	*pellegrini*	
nd4	0.0541	0.0479	0.0802	0.0521	0.0437	0.0514	0.0549
nd5	0.0599	0.0982	0.1014	0.0651	0.0773	0.0675	0.0782
nd6	0.0224	0.0300	0.0623	0.0163	0.0283	0.0321	0.0319
cytb	0.0209	0.0239	0.0264	0.0417	0.0335	0.0193	0.0276
nd1	0.0278	0.0329	0.0338	0.0503	0.0573	0.0391	0.0402
nd2	0.1114	0.0807	0.0818	0.0596	0.0820	0.0550	0.0784
cox1	0.0251	0.0117	0.0350	0.0255	0.0430	0.0312	0.0286
cox2	0.0387	0.0140	0.0261	0.0431	0.0158	0.0121	0.0250
atp8	0.0335	0.0000	0.1420	0.0000	0.0000	0.0278	0.0339
atp6	0.0483	0.0455	0.0682	0.0508	0.0481	0.0308	0.0486
cox3	0.0361	0.0000	0.0000	0.0000	0.0096	0.0000	0.0076
nd3	0.0253	0.0420	0.0512	0.0000	0.0209	0.0369	0.0294
nd4l	0.0235	0.0286	0.0000	0.0000	0.0000	0.0000	0.0087
**Average**	0.0405	0.0350	0.0545	0.0311	0.0353	0.0310	
