# Supplementary figures and images for: Characterization of the Complete Mitochondrial Genome of the Elongate Loach and Its Phylogenetic Implications in Cobitidae
Source: Animals (Basel). 2023 Dec 13;13(24):3841. doi: 10.3390/ani13243841 (PMC10740543; doi:10.3390/ani13243841)

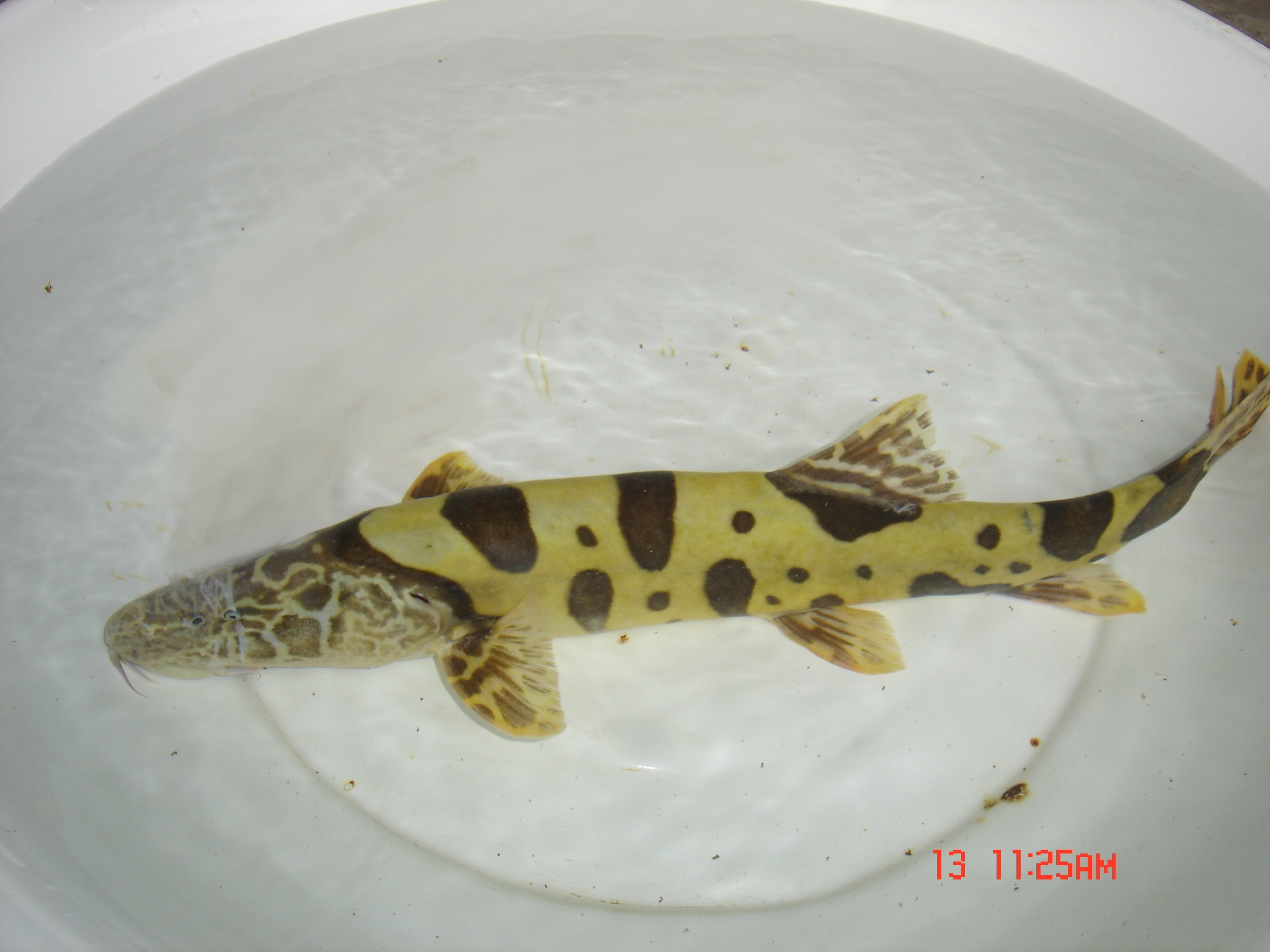

Supplement: Supplementary file 1 [file animals-13-03841-s001.zip › Elongate loach.jpg]
